# Nitric Oxide, an Essential Intermediate in the Plant–Herbivore Interaction

**DOI:** 10.3389/fpls.2020.620086

**Published:** 2021-01-08

**Authors:** Ana Arnaiz, Irene Rosa-Diaz, Maria C. Romero-Puertas, Luisa M. Sandalio, Isabel Diaz

**Affiliations:** ^1^Centro de Biotecnologia y Genómica de Plantas, Instituto Nacional de Investigación y Tecnología Agraria y Alimentaria (INIA), Universidad Politécnica de Madrid, Madrid, Spain; ^2^Department of Biochemistry and Molecular and Cellular Biology of Plants, Estación Experimental del Zaidín, CSIC, Granada, Spain; ^3^Departamento de Biotecnología-Biología Vegetal, Escuela Técnica Superior de Ingeniería Agronómica, Alimentaria y de Biosistemas, Universidad Politécnica de Madrid, Madrid, Spain

**Keywords:** nitric oxide, phytophagous arthropods, plant defenses, reactive nitrogen species, signaling molecules

## Abstract

Reactive nitrogen species (RNS), mainly nitric oxide (NO), are highly reactive molecules with a prominent role in plant response to numerous stresses including herbivores, although the information is still very limited. This perspective article compiles the current progress in determining the NO function, as either a signal molecule, a metabolic intermediate, or a toxic oxidative product, as well as the contribution of molecules associated with NO metabolic pathway in the generation of plant defenses against phytophagous arthropods, in particular to insects and acari.

## Introduction

Plants are in constant struggle with a variety of biotic stresses in nature that limit their survival. Among them, phytophagous arthropods are one of the most devastating groups. These herbivores employ specialized feeding modes to obtain nutrients causing leaf defoliation, chlorosis, biomass destruction, growth delay, and even worse consequences under severe infestations leading to an important negative impact in crop yields. Plants have developed sophisticated protection strategies against herbivore combining constitutive and inducible defenses, as the result of their long coexistence during the last 100 million years (Santamaria et al., 2013, 2018a). While constitutive defenses are constantly present, inducible ones are just activated in response to a specific threat, being their nature and mechanism of action directly targeted to the precise feeder and dependent on the plant species and developmental stage. The induction of defenses starts when plasma membrane-specific receptors (pattern recognition receptors, PRRs) recognize conserved herbivore-associated molecular patterns (HAMPs), microbe-associated molecular patterns (MAMPs) derived from herbivore symbionts, or damage-associated molecular patterns (DAMPs) linked to the herbivore injury. The perception of these molecular patterns promotes downstream short-term responses, first at the membrane level (potential depolarization, Ca^2+^ influxes, etc.), followed by the generation of reactive oxygen and/or nitrogen species (ROS and RNS) as signaling molecules, the activation of kinase cascades, and the synthesis of hormones to finally regulate the expression of defense genes (Fürstenberg-Hägg et al., 2013; Santamaria et al., 2018a). These cues prompt a set of defense events known as pattern-triggered immunity (PTI), by activating signal transduction pathways to synthesize defense metabolites (Jones and Dangl, 2006; Zipfel, 2014; Santamaria et al., 2018a). Alternatively, plant intracellular receptors identify herbivore molecules, elicitors or effectors, that selectively can either trigger or compromise plant immunity altering the defense machinery. This additional response, termed effector-triggered immunity (ETI), is considered an amplified reaction of the PTI (Tsuda and Katagiri, 2010). Early responses take place within minutes to hours after herbivore detection to then induce late-term responses whose products include defensive molecules with toxic, anti-nutritive, deterrent, or repellent properties and volatiles to attract natural enemies of the phytophagous pest (Santamaria et al., 2018a; Stahl et al., 2018; Erb and Reymond, 2019). The whole process is under the regulation of a complex hormonal crosstalk between jasmonic acid (JA), salicylic acid (SA), and ethylene (ET), besides other phytohormones. The known antagonistic relation between SA and JA allows a fine-tune regulation of the defense process (Erb et al., 2012; Schmiesing et al., 2016). Generally, JA-depending pathway is activated by chewing insects, whereas SA regulates responses induced by sucking-feeders (Bari and Jones, 2009; Pieterse et al., 2012), and a balance between JA/SA modulates defenses against sucking mites (Wei et al., 2014; Zhurov et al., 2014; Santamaria et al., 2020a).

Despite all the information available about the plant defense against arthropods, our knowledge on oxidative and particularly on nitrosative signaling is poorly understood. Levels of ROS and RNS, mainly hydrogen peroxide (H_2_O_2_) and nitric oxide (NO), increase during insect and acari infestation, and the redox status balance in the cell determines their function since moderate ROS/RNS concentrations differentially sense defense signaling, but an excess of oxidative stress produces chemical oxidation and induces programmed cell death (Foyer and Noctor, 2005; Bittner et al., 2017; Santamaria et al., 2017, 2018b).

## No Metabolism

**FIGURE 1:**
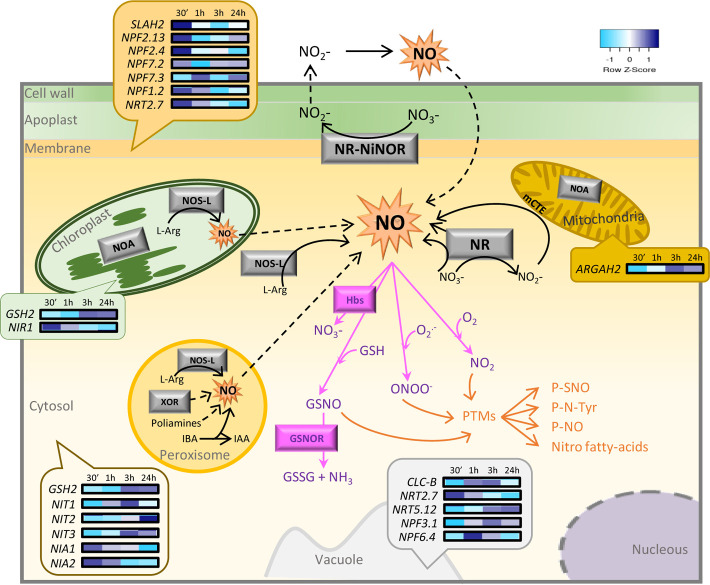
Schematic overview of NO sources and pathways in a plant cell and a heatmap of NO-associated genes expressed in the subcellular locations of *A. thaliana* after spider mite feeding. The diagram shows the main sources and pathways of NO (black arrows) including both oxidative and reductive pathways, the main scavengers (pink arrows) including superoxide ion, GSH, and hemoglobins, and the main NO mechanisms of action (orange arrows). Discontinued lines represent the mechanisms not experimentally demonstrated. A heatmap showing transcriptomic data of NO-associated genes from *A. thaliana* at different infestation times (30 min, 1, 3, and 24 h) with *T. urticae* is comprised within bubbles, positioned over the subcellular compartment where genes are expressed according to SUBA predictions, with a score ≥0.5. IAA, indole-3-acetic acid; IBA, indole-3-butyric acid; GSH, glutathione; GSNO, S-nitrosoglutathione; GSNOR, S-nitrosoglutathione reductase; Hbs, hemoglobins; L-Arg, L-arginine; mETC, mitochondrial electron transport chain; NR, nitrate reductase; NO, nitric oxide; NOS-L, nitric oxide synthase-like; NOA, NO-associated protein; P-NO, nitrosylated protein; P-N-Tyr, nitrated protein; P-SNO, S-nitrosylated protein; PTMs, post-translational modifications; XOR, xanthine oxidoreductase.

Nitric oxide is clearly recognized as an intra- and intercellular signaling molecule involved in the regulation of a huge range of plant processes ranging from development to resistance and defense responses to biotic and abiotic stresses (Sanchez-Vicente et al., 2019). Two pathways coexist in plants to produce NO, reductive and oxidative ones, involving nitrite and arginine as substrates, respectively (Leon and Costa-Broseta, 2020; Figure 1). Within reductive pathways, NO production arises by both enzymatic and non-enzymatic reactions and is usually dependent on oxygen and NO_2_^–^ concentrations. Nitrate reductase (NR), a multifunctional cytoplasmic enzyme, whose main function is nitrate assimilation to produce NO_2_^–^ in a NADPH-dependent way (Campbell, 2001), also shows nitrite reductase (NR) activity, although this represents only 1% of its reductase ability under normal conditions (Yamasaki and Sakihama, 2000; Rockel et al., 2002; Astier et al., 2019). NO production through the action of NR has been demonstrated using different approaches. The mitochondrial electron transport chain (mETC) under anaerobic/hypoxic conditions and the xanthine dehydrogenase–oxidase under anaerobic conditions or phosphate deficiency may also produce NO (Wang et al., 2010; Gupta et al., 2011; Cantu-Medellin and Kelley, 2013). On the other hand, under specific environmental conditions, such as low pH and high concentrations of NO_3_^–^, non-enzymatic reduction into NO takes place (Wendehenne et al., 2001; Bethke et al., 2004; Stöhr and Stremlau, 2006; Fancy et al., 2017).

The oxidative pathway involves the activity of specialized enzymes as the nitric oxide synthases (NOSs), which oxidize L-arginine to form L-citrulline and NO, and they are well characterized in mammals (Alderton et al., 2001). However, controversial results about this activity have been shown in plants. Bioinformatics approaches have shown no NOS gene/protein in higher plants (Jeandroz et al., 2016; Hancock, 2019), excluding some algae (Foresi et al., 2015), and the typical mammalian NO–cGMP signaling pathway has been also questioned (widely reviewed in Astier et al., 2019). Nevertheless, NOS-like activity has been extensively described in plants by the use of NOS inhibitors and even by heterologous expression of mammalian NOS (Zeidler et al., 2004; Ali et al., 2007; Astier et al., 2018), and the denomination “NOS-like” is adopted for this activity.

Once synthesized, NO is highly reactive, and there are three main types of molecules that react with NO: ROS, glutathione (GSH), and metals (Romero-Puertas and Sandalio, 2016). NO rapidly reacts when present, with the radical superoxide (O_2_^–^) generating peroxynitrite (ONOO^–^), which is one of the most potent oxidant molecules in the cell leading to lipid peroxidation, protein nitration (Ischiropoulos and al-Mehdi, 1995; Radi, 2004), oxygenated forms of cysteine (Cys) residues (sulfenic, sulfinic, and sulfonic acids), and S-glutathionylation (Martínez-Ruiz et al., 2013). ONOO^–^ has been shown to be produced under different stress conditions in plants (Romero-Puertas et al., 2007; Arasimowicz-Jelonek and Floryszak-Wieczorek, 2019). NO can also react with lipid peroxyl radical (LOO⋅) to produce nitro-fatty acids that are related to plant development and plant response to abiotic stress (Rubbo, 2013; Mata-Perez et al., 2017). Besides, the reaction of NO with GSH produces nitrosoglutathione (GSNO), which is considered an endogenous NO reservoir (Noctor et al., 2012) and acts as an S-nitrosylating agent. GSNO is metabolized by GSNO reductase (GSNOR) to transform GSNO into glutathione disulfide (GSSG) and ammonia. Thus, GSNOR controls intracellular levels of GSNO and NO and, therefore, plant responses under different conditions (Liu et al., 2001; Yun et al., 2016). On the other hand, globins are proteins able to metabolize NO producing NO_3_^–^ (Perazzolli et al., 2004; Becana et al., 2020), and consequently, these proteins can control NO levels by detoxification or through post-translational modification (PTM) reactions (Perazzolli et al., 2006; Figure 1).

## No Mechanism of Action: Crosstalk With ROS and H_2_S

Nitric oxide reactivity leads to its main mechanism of action being PTM of proteins, which are carried out by a series of RNS produced by the reaction of NO with other free radicals as described before. PTMs best studied in plants are: (i) S-nitrosylation/S-nitrosation, referred to the formation of a nitrosothiol group in cysteines, with more than thousand targets described in plants, although a small number have been characterized (Sanchez-Vicente et al., 2019; Sandalio et al., 2019); (ii) nitration, being mainly studied the addition of a nitro group to Tyr side chain, with more than hundred targets described and only a dozen characterized (Rubbo and Radi, 2008; Sanchez-Vicente et al., 2019), and (iii) nitrosylation of transition metals, with the formation of complex bonds to heme groups (Martinez-Ruiz and Lamas, 2009), scarcely studied in plants. NO-dependent PTMs result in the induction of different physiological responses and/or signaling processes as alteration of gene expression, metabolic changes, and phytohormone signaling. Furthermore, NO may regulate other signaling pathways, such as phosphorylation, oxidation, and ubiquitinylation (Cui et al., 2018; Leon and Costa-Broseta, 2020; Lindermayr et al., 2020). Therefore, the ability to regulate virtually all processes in the plant makes NO a do it all molecule (Delledonne, 2005).

Post-translational modification regulation of proteins is quite complex, however, due to the synergistic and antagonistic interplays between the different PTMs (Sandalio et al., 2019). Overlapping of different PTMs on the same protein is very often and follows common pattern in different species, which demonstrate the importance of multilevel PTM regulation in cell metabolism (Duan and Walther, 2015). NO crosstalk with other signaling molecules, such as the well-known ROS and the lesser-known sulfide (H_2_S), leads to an interplay between redox-dependent PTMs being targets the sulfur-containing amino acids, such as cysteine. Thus, the first step in Cys oxidation is S-nitrosylation while the main ROS involved in signaling, H_2_O_2_, leads Cys to the following steps, its reversible oxidation to sulfenic acid (–SOH; sulfenylation) and sulfinic acid (–SO_2_H; sulfinylation). Excessive ROS accumulation gives rise to the irreversible sulfonic acid (–SO_3_H; sulfonylation) derivative (Young et al., 2019). S-nitrosylation, sulfenylation, sulfinylation, and intra- and intermolecular disulfide bond formations are rapid and reversible mechanisms to regulate protein function, stability, and location of proteins (Sandalio et al., 2019; Young et al., 2019). Due to their transient nature, these sulfur modifications, which can be reversibly reduced by thioredoxin and glutaredoxin pathways, are regarded as redox switches, giving rise to rapid finely tuned regulation of metabolic pathways and signaling processes (Sandalio et al., 2019; Young et al., 2019). H_2_S, involved in regulating various processes essential for plant survival, has been demonstrated recently to be a signaling molecule in the same degree of NO and H_2_O_2_ in plant systems (Gotor et al., 2019; Hancock, 2019). The mechanism of action of H_2_S is related with its high affinity for metals from metalloproteins, but it also can oxidize Cys thiol groups to persulfide groups (R-S-SH) promoting covalent PTMs termed persulfidation, which could play a protective role for thiols against oxidative damage (Gotor et al., 2019). Interestingly, RNS and ROS levels are regulated by the interplay between ROS-, H_2_ S-, and NO-dependent PTMs. Curiously, S-nitrosylation prevents ROS-dependent oxidative damage to several proteins involved in the Calvin–Benson cycle, probably by inducing conformational changes in specific proteins (Tanou et al., 2012). Crosstalk between NO and H_2_S has been reported in acclimation processes in citrus plants (Molassiotis et al., 2016). On the other hand, antagonistic interplay between protein Tyr nitration and phosphorylation competing for the same Tyr sites has been reported, interfering with different cellular processes, such as cell signaling *via* MAP kinase cascades (Arasimowicz-Jelonek and Floryszak-Wieczorek, 2019). Although several proteins have been shown as targets of NO-dependent PTMs under different stress conditions, in particular, plant–herbivore interaction is a field that needs to be better explored.

## No in Plant–Herbivore Interactions

Some publications have described the rapid accumulation and participation of NO as a common feature to insect-infested plants (Table 1). Different arthropods including hemipteran (Smith and Boyko, 2007; Moloi and van der Westhuizen, 2009; Liu et al., 2011; Mai et al., 2014; Wozniak et al., 2017; Li et al., 2019; Xu et al., 2020) and lepidopteran species (Arimura et al., 2008; Bricchi et al., 2010) cause a rapid and transient increase of NO levels in insect-damaged tissues. However, its physiological significance remains to be established. NO has not been linked to Vm depolarization as H_2_O_2_ has, but it has been related to Ca^2+^ homeostasis and cGMP signaling (Wu and Baldwin, 2009; Misra et al., 2011). Thus, it could exert its biological function through the mobilization of secondary messengers or by the modulation of protein kinase activity. NO interacts with ROS and phytohormones (Mur et al., 2013) and, in consequence, may indirectly act as regulator of the gene expression. In addition, the PTM of proteins mediated by NO, described above, may have potential regulatory effects in plant defense against herbivores as it does toward plant pathogens (Mur et al., 2006; Martinez-Medina et al., 2019).

**TABLE 1:** Participation of NO and NO-related enzymes in the plant defenses against phytophagous insects.

Species		Description	Effects	References
Plant	Herbivore			
Several species	Several aphids	Infestation	Accumulation of NO	Smith and Boyko (2007)
*Phaseolus lunatus*	*Spodoptera littoralis*	Infestation	Accumulation of NO	Arimura et al. (2008)
*Triticum aestivum*	*Diuraphis noxia*	Infestation	Accumulation of NO	Moloi and van der Westhuizen (2009)
*Phaseolus lunatus*	*Spodoptera littoralis*	Infestation	Accumulation of NO	Bricchi et al. (2010)
*Oryza sativa*	*Nilaparvata lugens*	Infestation	Accumulation of NO	Liu et al. (2011)
			Induction of NOS activity	
*Nicotiana attenuat a*	*Manduca sexta*	Infestation of *GSNOR* knock-down	Reduction of JA and ET	Wünsche et al. (2011a)
			Reduction of trypsin proteinase inhibitor activity and diterpene glycosides	
*Nicotiana attenuata*	*Manduca sexta*	Infestation of *NOA1* Knock-out	Reduction of carbon-based defensive molecules	Wünsche et al. (2011b)
*Pisum sativum*	*Acyrthosiphon pisum*	Infestation	Accumulation of NO, H_2_O_2_, JA, SA, and ET	Mai et al. (2014)
*Pisum sativum*	*Acyrthosiphon pisum*	Infestation and application of NO donors	Accumulation of NO	Wozniak et al. (2017)
			Induction of defensive molecules (phenylalanine ammonia lyase and pisatin)	
*Nicotiana tabacum*	*Manduca sexta*	Infestation	Induction of nitrogen-derived defensive metabolites (alkaloids)	Campbell and Vallano (2018)
			Decrease in foliar N-uptake	
*Oryza sativa*	*Nilaparvata lugens Sogatella furcife ar*	Infestation of *MAPK20-5* Knock-out	Accumulation of NO and ET	Li et al. (2019)
*Nicotiana tabacum*	*Bemisia tabaci*	Infestation of NOA1 knock-out	Accumulation of NO	Xu et al. (2020)
			Suppression of JA-dependent defenses	

In seedling leaves of pea (*Pisum sativum*), Mai et al. (2014) described the convergence of NO and H_2_O_2_ accumulation with the induction of JA, ET, and SA, hormones that sequentially appeared within the first 24–96 h after the aphid *Acyrthosiphon pisum* feeding. The simultaneous generation of hormones and free radicals at the same time points suggested a synergistic defense action in pea plants to aphid infestation. Moreover, the application of exogenous NO donors (NO, GSNO, and SNP, sodium nitroprusside) to pea plants infested with *A. pisum* revealed the induction of defense reactions leading to a deterrent result on the pea aphid feeding and the reduction in its population growth (Wozniak et al., 2017). A side effect of SNP treatment is the release of cyanide, a potent respiratory poison with a deterrent effect on phytophagous arthropods who try to elude it or detoxify (Pentzold et al., 2014; Keisham et al., 2019). Campbell and Vallano (2018) analyzed the effects of atmospheric NO_2_ leaf uptake on tobacco (*Nicotiana tabacum*) metabolism and its impact in the tobacco responses to the lepidopteran *Manduca sexta*. Results showed that the foliar assimilation of NO_2_ increased the nitrogen-derived defensive metabolites, particularly of some alkaloids, and diminished insect feeding and growth. To avoid this defense mechanism, herbivore modified somehow the plant capacity to absorb the reactive nitrogen, prompting a decrease in foliar nitrogen uptake and limiting the concentration of metabolites in leaves. Moreover, accumulating evidences indicate that an interactive fashion of phytohormones and NO regulates guard cell ABA-signaling and stomatal closure, which restricts the foliar uptake of NO_2_ (Sun et al., 2019). In turn, only few available reports have demonstrated the function of enzymes and other molecules associated with NO metabolic pathway in the generation of plant defenses to pests. Li et al. (2019) showed that the NO production in rice (*Oryza sativa*) plants was associated with their responses to *Nilaparvata lugens* infestation, in both susceptible and resistant cultivars. The rice planthopper feeding induced the activity of the NOS-like enzyme only in the susceptible cultivar, whereas no significant alterations of the NR enzymatic activity were observed, in none of the two rice-infested cultivars. These results suggested the active role of NOS in rice defense mediated by NO. Likewise, Wünsche et al. (2011a) examined the function of the GSNOR enzyme in the plant–herbivore interaction by knocking-down GSNOR in *Nicotiana attenuata* plants. A decrease in JA and ET levels in the silenced plants was observed concomitant to an elevated susceptibility to *M. sexta* attack. Accordingly, the *GSNOR*-silenced tobacco plants showed a significant reduction of the trypsin proteinase inhibitor activity and in the diterpene glycosides content, both considered secondary defensive metabolites dependent on the JA derivatives. Wünsche et al. (2011b) also proved that the *N. attenuata* NO-associated protein 1 (NOA1) was required for the accumulation of JA and JA-Ile and the generation of defenses against *M. sexta. NOA1*-silenced tobacco plants compromised the production of most of the carbon-based defensive compounds while the synthesis of nitrogen-rich defense metabolites was not altered. These results were probably due to the role of NOA1 in plant chloroplast functions and in the allocation of carbon resources within phenylpropanoid pathway (Wünsche et al., 2011b). Very recently, Xu et al. (2020) have demonstrated that the hemipteran *Bemisia tabaci* infestation activated NO signaling in tobacco, leading to suppression of JA-dependent defenses and improving nymph performance. Additionally, they have confirmed the NOA1 involvement in the JA-mediated responses to *B. tabaci*.

The mechanism by which NO mediates the enhancement of plant defenses against pests is still poorly studied, but a recent publication by Li et al. (2019) has linked a mitogen-activated protein kinase, OsMAPK20-5, to NO production in *N. lugens*-infested rice plants. The *OsMAPK20-5* gene expression was up-regulated by female adult feeding, which presumably could be a response to oviposition. Surprisingly, the levels of NO and ET increased after insect feeding in the *OsMAPK20-5*-silenced plants and consequently improved rice resistance to brown planthopper and oviposited eggs. According to the authors, OsMAPK20-5 could enable rice plants to control excessive hyperaccumulation of NO and ET and thereby to prevent autotoxicity. Importantly, in field trials, *MAPK20-5*-silenced rice lines displayed a wide protection not only to the *N. lugens* but also to the white-backed planthopper *Sogatella furcifera*. Therefore, NO could mediate defense responses in plants against pests acting as a signal molecule, a metabolic intermediate, or a toxic oxidative product.

Since no information on the NO’s role in the interplay between plant and phytophagous acari was available, we did a search of NO-related genes in the RNA sequencing of *Arabidopsis thaliana* in response to the spider mite *Tetranychus urticae* after 30 min, 1, 3, and 24 h of feeding (Supplementary Material; Santamaria et al., 2020b). Nineteen NO-associated genes, mainly encoding nitrate transporters, NRs, and nitrilases, were differentially expressed at different time points of infestation. Nitrate transporters showed different expression patterns based on their subcellular *in silico* location. Generally, those transporters located at the cytoplasmic membrane were rapidly induced by mite infestation, followed by the ones located at the vacuole. *NIA1* and *NIA2* genes that encode RNS were highly up-regulated at 30 min after mite feeding but were repressed at 24 h. Glutathione synthetase 2 (*GSH2*) gene putatively located at the chloroplast and cytosol and arginine amidohydrolase 2 (*ARGAH2*) gene product located at the mitochondria presented the opposite expression pattern being induced at longer infestation time (Figure 1). These differential expression profiles are according to the consecutive steps of plant defense to mite attack since after mite perception, signaling is first activated at the cell membrane level and then transmitted through the cytosol to the rest of the organelles to finally induce the expression of defensive genes. In addition, the identified genes were classified into five different over-expressed categories based on their Gene Ontology (GO) biological function, all of them related to RNS metabolic processes (Supplementary Table 1). These data suggested their functional significance during *T. urticae* infestation. Further studies are needed to clarify the NO and NO metabolic pathways in the plant defenses against acari feeders.

In conclusion, the current information on how plant responses are regulated by NO and NO-related molecules constitutes still a set of unknown events to be explored, particularly, in the plant–acari interplay. An advanced understanding of the NO function in plant–herbivore interactions will be a strong tool to enhance crop performance and potentially lead to biotechnological approaches for pest control in agricultural systems.

## Data Availability Statement

The original contributions presented in the study are included in the article/Supplementary Material, further inquiries can be directed to the corresponding author.

## Author Contributions

ID conceptualized the manuscript content. AA and IR-D performed the experiment. ID, AA, MR-P, and LS wrote different sections of the first draft. All authors contributed to the final version of the manuscript.

## Conflict of Interest

The authors declare that the research was conducted in the absence of any commercial or financial relationships that could be construed as a potential conflict of interest.
