# Supplementary material for: Nitric Oxide, an Essential Intermediate in the Plant–Herbivore Interaction
Source: Front Plant Sci. 2021 Jan 8;11:620086. doi: 10.3389/fpls.2020.620086 (PMC7819962; doi:10.3389/fpls.2020.620086)
Supplement: Supplementary file 1 [file Data_Sheet_1.docx]

**Supplementary methods**

A search of NO-related genes was done in the RNAseq data of *Arabidopsis thaliana* in response to the spider mite *T. urticae* at different time points (30 min, 1, 3 and 24 h) of feeding (Santamaria et al., 2020b). Differentially expressed genes involved in NO pathway were identified and those showing a p-adjusted value < 0.05 and a log2Ratio (fold change) higher than 1 were selected. These genes were differentially expressed at least in one of the infestation times.

Gene Ontology analysis (GO) using Gene Ontology Consortium tools (http://www.geneontology.org) was performed in order to find genes related with NO pathway. GO selected categories passes Fisher’s exact test (GraphPad Prism 6 software) with significant P value summary: P, 0.001 (***) and P, 0.0001 (****). Then, a manually selection based on the literature, was applied. NO-associated genes are represented in Figure 1 as a heat map, showing de row Z-score. Z-score allows the categorization of each gene by its expression level. Analysis of chosen data was conducted by Python 3.9, NumPy and Seaborn libraries. Additionally, to determine protein locations, the SUBcellular location database for Arabidopsis proteins (SUBA4: <http://suba.live>) were used (Hooper et al. 2017). All the represented localizations have a SUBAcon score ≥0.5

Hooper, C. M., Castleden, I., Tanz, S. K., Aryamanesh, N., and Millar, A. H. (2017). SUBA4: the interactive data analysis centre for Arabidopsis subcellular protein locations. [*Nucleic Acids Res.*](https://academic.oup.com/nar/article-lookup/doi/10.1093/nar/gkw1041) 45, D1064-D1074. doi: 10.1093/nar/gkw1041

|  | | |  | |  | **Fisher test significance** |
| --- | --- | --- | --- | --- | --- | --- |
| **Go ID** | **Term** | | | **User data** | **Genome data** |  |
| *GO:0010243* | | Response to organonitrogen compound | | 97 | 166 | **** |
| *GO:2001057* | | Reactive nitrogen species metabolic process | | 15 | 46 | **** |
| *GO:1901698* | | Response to nitrogen compound | | 108 | 264 | **** |
| *GO:1901565* | | Organonitrogen compound catabolic process | | 41 | 164 | **** |
| *GO:0071941* | | Nitrogen cycle metabolic process | | 15 | 46 | **** |

**Suppl. Table 1: NO related genes classified by GO terms**. Fisher’s exact test (*, P, 0.05; **, P, 0.01; ***, P, 0.001; and ****, P, 0.0001) performed using DEGs from Santamaria et al 2020b and Arabidopsis genome genes that belong to each category.
